# CD49b, CD87, and CD95 Are Markers for Activated Cancer-Associated Fibroblasts Whereas CD39 Marks Quiescent Normal Fibroblasts in Murine Tumor Models

**DOI:** 10.3389/fonc.2019.00716

**Published:** 2019-08-05

**Authors:** David J. Agorku, Anne Langhammer, Ute Heider, Stefan Wild, Andreas Bosio, Olaf Hardt

**Affiliations:** ^1^Miltenyi Biotec GmbH, Bergisch Gladbach, Germany; ^2^HAN Master Programmes, HAN University of Applied Sciences, Nijmegen, Netherlands

**Keywords:** cancer-associated fibroblasts, normal fibroblasts, subcutaneous 4T1 mouse tumor model, subcutaneous CT26.WT mouse tumor model, CD39, CD87, CD49b

## Abstract

Fibroblasts are thought to be key players in the tumor microenvironment. Means to identify and isolate fibroblasts as well as an understanding of their cancer-specific features are essential to dissect their role in tumor biology. To date, the identification of cancer-associated fibroblasts is widely based on generic markers for activated fibroblasts in combination with their origin in tumor tissue. This study was focused on a deep characterization of the cell surface marker profile of cancer-associated fibroblasts in widely used mouse tumor models and defining aberrant expression profiles by comparing them to their healthy counterparts. We established a generic workflow to isolate healthy and cancer-associated fibroblasts from solid tissues, thereby reducing bias, and background noise introduced by non-target cells. We identified CD87, CD44, CD49b, CD95, and Ly-6C as cancer-associated fibroblast cell surface markers, while CD39 was identified to mark normal fibroblasts from healthy tissues. In addition, we found a functional association of most cancer-related fibroblast markers to proliferation and a systemic upregulation of CD87, and CD49b in tumor-bearing mice, even in non-affected tissues. These novel markers will facilitate the characterization of fibroblasts and shed further light in their functions and implication in cancer progression.

## Introduction

Several aspects of tumor growth and progression have been associated with a distinct type of activated fibroblasts, often termed cancer-associated fibroblasts (CAFs) (1, 2). It has been reported that CAFs can induce angiogenesis by activating endothelial cells, and pericytes in the tumor microenvironment (3, 4), that they can directly affect tumor cells by promoting stemness, and drug resistance (5), and support epithelial-to-mesenchymal transition (EMT) (6). Different regulatory molecules and traits have been identified that are closely associated with the reprogramming and metabolic changes that occur in cancer, many of which have been demonstrated to be affected by fibroblasts (7–10). Via secretion of paracrine factors, CAFs have been shown to regulate the recruitment of inflammatory cells (11). Fibroblasts represent a highly plastic cell type displaying a range of activation states. While normal fibroblasts (NFs) are small and thin spindle-shaped cells, cancer-associated fibroblasts are reported to be large, more branched and expressing stress fibers (1, 12). Phenotypically, CAFs share many features with activated fibroblasts, e.g., during wound healing, but have mainly been characterized, and detected by intracellular markers and based on patterns of secreted factors. Multiple markers have been used to identify CAFs, including CD44, α-smooth muscle actin (αSMA), fibroblast-activated protein (FAP), fibroblast-specific protein-1 (S100A4), neuron-glial antigen-2 (NG-2), and platelet-derived growth factor α, and β-receptor (PDGFRα and PDGFRβ) (5, 13, 14). However, most of these markers can also be detected in NFs, for example upon *in vitro* propagation based activation. Nevertheless, the role of those markers in CAFs is only poorly understood. Additionally, the expression of CAF-markers seems to be dependent on the tumor entity which complicates identification and also isolation strategies relying on the expression of defined cell-surface markers. Widely used and accepted markers such as αSMA are intracellular markers and therefore not suitable for identification and isolation of viable cells.

The central role of fibroblasts in the tumor microenvironment and cancer progression has been commonly recognized, however, the identification of markers specific for the cancer-associated phenotype of fibroblasts has not been achieved so far. Therefore, the aim of this study was the identification of CAF-specific cell surface markers in order to facilitate identification and isolation of this cell type from mouse tumor models. As a prerequisite for the identification of cancer-related alterations in fibroblasts, we employed normal fibroblasts isolated from healthy tissues as matched reference population, a highly important control lacking in key publications (5, 15–20). This allowed for the identification of novel cell surface markers associated with normal or cancer-associated fibroblasts. Moreover, we were able to show that the expression of those markers correlates to the activation states of fibroblasts.

## Materials and Methods

### Ethical Guidelines for Animal Research

All experiments were performed in compliance with European and German guidelines for the care of laboratory animals and were approved by the ethical committee on animal care and use in North Rhine-Westphalia (approval number: 84_02.04.2014.A325).

### Mice

Six to eight week-old BALB/c female mice (Charles River) were used for tumor cell injection and for healthy mammary fat pad and skin. Subcutaneous inoculation was performed by injecting 10^6^ cells into the flanks of mice. Tumor growth was monitored by repetitive measurements with calipers and noted as width × length.

### Tissue Dissociation

Mouse tumor tissue was dissociated into a single cell suspension using the Tumor Dissociation Kit, mouse in combination with the gentleMACS Octo Dissociator with heaters (both Miltenyi Biotec) according to the manufacturer's instructions. Mouse mammary fat pads and skin tissue were dissociated together using Tumor Dissociation Kit, mouse in combination with the gentleMACS Octo Dissociator with heaters (both Miltenyi Biotec) according to manufacturer's instructions. Notably, a custom made protocol was run on the gentleMACS Octo Dissociator with heaters for skin and mammary fat pad from mice. In brief, the tissue was incubated for 1 h at 37°C and under constant rotation at 20 rpm, followed by the program 37C_h_TDK_1.

### Flow Cytometric Analysis

For flow cytometric analysis, single cell suspensions were stained with the indicated antibodies (see Materials and Methods and Tables S1, S2) according to the manufacturer's instructions and analyzed using the MACSQuant™ Analyzer (Miltenyi Biotec). Cells were stained at a density of 0.5–2 × 10^5^ cells per sample in 50 μL volume of PBS pH 7.2, 2 mM EDTA, and 0.5% BSA (PEB) buffer at 2–8°C for 10 min, followed by a washing step with PEB. Cell viability was assessed by Propidium Iodide (PI, 1 μg/mL, Miltenyi Biotec) prior to flow cytometric analysis. Analysis was performed using the MACSQuant Analyzer and data analysis using the MACSQuatify software (Miltenyi Biotec).

### Isolation of CD90.2 Expressing Fibroblasts

Normal or cancer-associated fibroblasts were isolated by magnetic activated cell sorting (MACS) based on CD90.2 expression. After dissociation fibroblasts were first pre-enriched and then isolated using the Tumor-associated Fibroblast Isolation Kit, mouse (Miltenyi Biotec) with LD- and MS-columns (Miltenyi Biotec), respectively. The isolation procedure was performed according to the manufacturer's instructions. Purity and yield were assessed by flow cytometric analysis using fluorochrome-conjugated CD90.2 antibodies, CD45 antibodies, and Labeling Check Reagent (all Miltenyi Biotec).

### Cell Culture

The mouse breast cancer cell line 4T1 and the colon carcinoma cell line CT26.WT were maintained in Dulbecco's modified Eagle's medium (DMEM, Biowest) supplemented with 10% fetal calf serum (FCS; Biochrom), 10 mM HEPES (Lonza), 2 mM L-glutamine (Lonza), and penicillin/streptomycin (Lonza). Cells were maintained at 37°C in a humidified 7.5% CO_2_ atmosphere.

CD90.2 expressing stromal cells were isolated by MACS from BALB/c mouse mammary fat pad and skin or 4T1 or CT26.WT tumors generated by subcutaneous injection into BALB/c mice (Charles River). Isolated primary cells were plated on Matrigel (Corning) coated tissue culture ware and grown in DMEM supplemented with 8% FCS, 10 mM HEPES, 2 mM L-glutamine, P/S, and 1x non-essential amino acids (NEAA, Lonza) at 37°C in a humidified 7.5% CO_2_ atmosphere.

### Flow Cytometry–Based Proliferation Analysis

To determine whether any of the identified surface markers are associated with proliferation, isolated primary cells were stained using CellTrace Violet (Life Technologies) according to the manufacturer's instructions, followed by cultivation as previously described. Briefly, cells were washed with PBS after isolation, incubated with 5 μM CellTrace Violet at room temperature for 20 min. Next culture medium as described above was added, cells were incubated for additional 5 min at room temperature, centrifuged at 300 × g for 5 min and then resuspended in culture medium. An aliquot of the cells was analyzed using the MACSQuant Analyzer and the rest of the cells was cultivated as described above.

### Statistical Analysis

All statistics and final plots were obtained using GraphPad Prism 6 software and all statistically evaluated experiments were performed in three replicates from at least two independent experiments. For comparison of three different groups (e.g., NFs, 4T1 CAFs, and CT26.WT CAFs), two-way ANOVA was used.

## Results

### Generic Isolation of Fibroblast Populations From Solid Tissues

**Figure 1:**
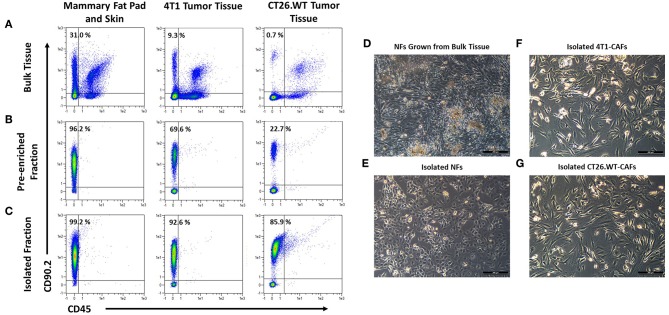
Isolation and cultivation of fibroblasts from solid tissues. **(A–C)** Flow cytometric analysis of cells before and after cell sorting. Fibroblasts, identified by expression of CD90.2 and absence of CD45, occurred at varying frequencies in different tissues **(A)**. Fibroblasts were pre-enriched by depletion of CD90.2-expressing leukocytes and other non-fibroblast populations **(B)**. Subsequently, CD90.2-expressing fibroblasts were labeled and magnetically separated from the pre-enriched fraction **(C)**. **(D–G)** Images of cultivated fibroblasts. Normal fibroblasts, grown from single cell suspension of bulk tissues showed an elongated, spindle-shaped morphology, typical for activated fibroblasts **(D)**, while fibroblasts isolated from healthy tissues showed the typical compact shape of normal fibroblasts **(E)**. Isolated cancer-associated fibroblasts showed a spindle shaped morphology with an overall elongated appearance **(F,G)**.

The aim of the study was the characterization of CAFs from solid mouse tumors, using NFs as matched control populations. In order to identify fibroblast populations in healthy tissues and tumors, we used antibodies recognizing the generic fibroblast marker CD90.2 (Thy-1.2). Thy-1 proteins are widely known markers for fibroblasts from different species and tissues (21–23). We established a method for CD90.2 (Thy-1.2) based isolation of fibroblasts which can be employed for the isolation of both, normal, and cancer-associated fibroblasts. Cells were isolated from dissociated solid tissues by immunomagnetic sorting. As subpopulations of murine leukocytes also express CD90.2 and the starting frequencies of fibroblasts can be very low, in particular in tumor tissues, a two step-procedure was employed to consistently achieve high purity target cells. Non-target cells such as leukocytes and erythrocytes were depleted, followed by a CD90.2-postive selection (Figures 1A–C). Isolated fibroblasts showed a typical morphology *in vitro*: NFs had a small and compact shape, while CAFs were spindle-shaped with an overall elongated appearance (Figures 1D–G). Of note, NFs grown out of heterogenous bulk cell suspensions showed a CAF-like morphology (Figure 1E), suggesting an activated state and clearly demonstrating the advantage of working with isolated cells as those were not activated *in vitro*, thus more closely resembling the *in vivo* state of the cells. In addition, by isolating the target cells a reduction of a bias in downstream analyses by contaminating cell populations is achieved.

### Identification of Differentially Expressed Cell Surface Markers Among NFs and CAFs

**Figure 2:**
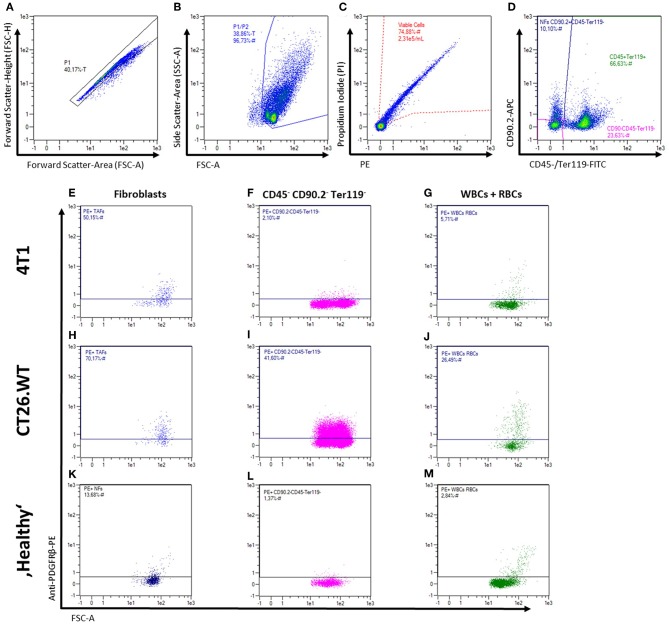
Flow cytometric analysis of fibroblasts from solid tissues. **(A–M)** Representative gating strategy and read-out for analysis of cell surface marker expression. First, cells are gated in a FSC-A vs. FSC-H plot to exclude doublets **(A)**, followed by exclusion of debris in a FSC-A vs. SSC-A plot **(B)**, and dead cell exclusion on a PE-A vs. PI-A plot **(C)**. Fibroblasts, leukocytes (WBCs), erythrocytes (RBCs), and triple-negative cells were then gated in a plot of FITC-A (CD45 and Ter119) vs. APC-A (CD90.2; **D**) for independent analysis of the different populations and mean fluorescence intensity levels **(E–M)**. Representative expression patterns of PDGFRβ (CD140b) are shown for 4T1 and CT26.WT tumor cells as well as for bulk skin and mammary fat pad cells from BALB/c mice.

Fibroblasts from subcutaneously grown 4T1 and CT26.WT tumors were compared to normal BALB/c mouse skin and mammary fat pad fibroblasts by flow cytometry-based marker screening. The screening was performed using 233 PE-conjugated monoclonal or REAfinity antibodies (Table S1). In order to identify fibroblasts within the bulk cells, the samples were co-stained with CD90.2 as generic fibroblast marker as well as CD45 and anti-Ter119 as markers to exclude CD90.2 positive non-fibroblasts (i.e., leukocytes and erythrocytes, respectively). Figures 2A–D shows the gating strategy used for all tissues analyzed in the initial screening, as well as the readout of the analysis with the expression level of each marker (Figures 2E–M). The initial screenings revealed 87 markers expressed by 4T1-derived fibroblasts, 87 markers expressed by CT26.WT-derived fibroblasts, and 64 markers expressed on normal fibroblast derived from skin and mammary fat pad of healthy mice. By comparing the markers found to be expressed on the distinct fibroblast populations of the respective tissues with the expression patterns of the leukocyte/erythrocyte and the triple-negative population (CD90.2neg CD45neg Ter119neg), fibroblast-specific markers of the respective tissues were identified (Figures 2E–M). 4T1 tumor-derived fibroblasts showed 16 (out of 87) fibroblast-specific markers, CT26.WT tumor-derived fibroblasts exclusively expressed 9 (out of 87) surface markers, and normal fibroblasts from skin and mammary fat pad showed expression of 9 (out of 64) specific markers. To identify markers differentially expressed among normal and tumor-derived fibroblasts, the markers found to be expressed in the CD90.2pos CD45neg Ter119neg fibroblast populations of the different parental tissues were compared (see Supplementary Figure 1). Among those markers, 12 surface molecules were exclusively found on NFs, while 27 markers were only found on both tumor-derived fibroblast populations. Next to the CAF-related markers, 9 markers specific for the 4T1 tumor-derived fibroblast population and 12 markers specific for the CT26.WT tumor-derived fibroblast population were identified. Based on these results, a smaller panel of 62 markers was further analyzed and validated (Table S2).

### Validation of Candidate Markers on Isolated Fibroblasts

To validate the most important markers as determined in the initial screening, fibroblasts derived from healthy BALB/c mouse skin, healthy BALB/c mammary fat pad, 4T1, and CT26.WT derived tumors were isolated using CD90.2 immunomagnetic sorting followed by flow cytometry based analysis. The expression of 29 markers could be validated on isolated fibroblasts, while no expression of the remaining 33 markers could be detected after isolation. Hierarchical clustering of the validation data (see Supplementary Figure 2) suggested a close relation of the analyzed CAF populations, while NFs clustered more distinctly. The molecules EphA2, GARP, MHC class II, Rae-1a/b/g, CD80, CD87, CD106, CD123, and CD150 were found to be only expressed on 4T1- and CT26.WT-derived fibroblasts. Yet, only CD87, CD106, and Rae-1a/b/g marked a clear subpopulation (>5 % of fibroblasts) in both tumor types, suggesting a cancer-associated regulation rather than a tumor type-related effect. CD39 was clearly overrepresented on normal fibroblasts isolated from “healthy” tissues. In addition CD95, CD140a, and CD140b were found on a minor fraction of NFs but overrepresented on CAFs. Taken together, CD39 was identified as a potential marker for NFs while the markers CD87, CD106 and Rae-1a/b/g were found to be potential CAF-specific markers. However, CD106, and Rae-1a/b/g were only expressed on a small fraction of CAFs, reducing their value as general CAF markers. Additionally, CD95 showed a marked difference in frequency among normal and cancer-associated fibroblasts. Surprisingly, Ly-6C was found to be expressed on a substantial fraction of all fibroblast populations, although Ly-6C has recently been introduced and discussed as specific marker for a CAF subpopulation termed inflammatory CAFs (iCAFs).

### Cultured CAFs Resemble an *ex vivo* Phenotype While Cultivation of NFs Induces Expression of CAF-Associated Markers

**Figure 3:**
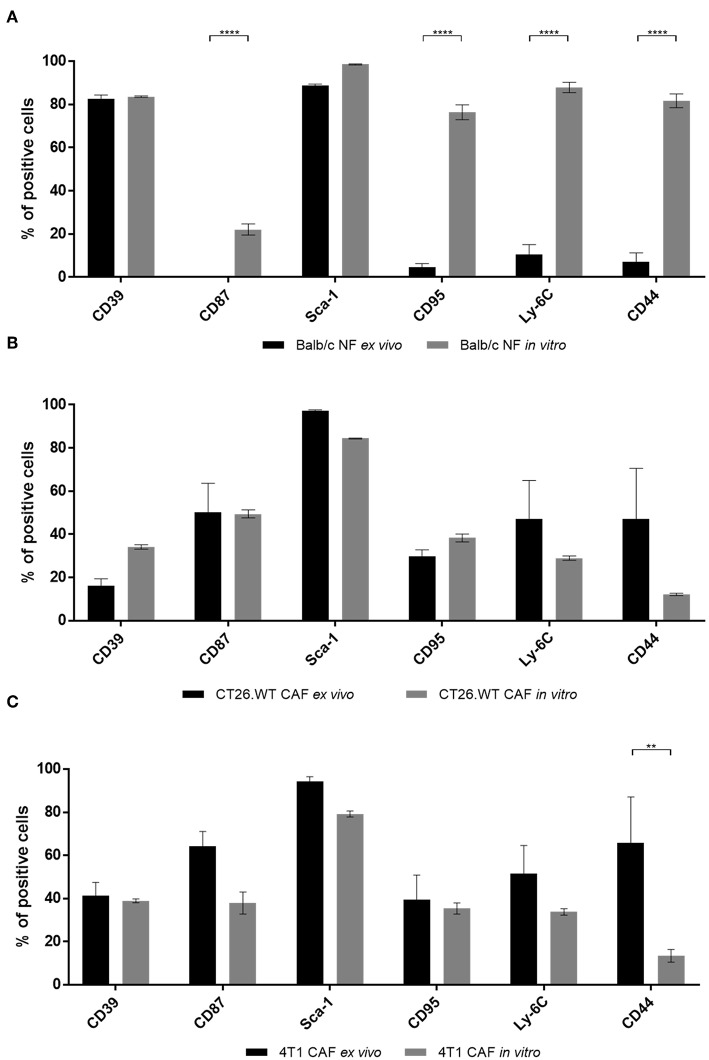
Comparison of *ex vivo* vs. *in vitro* phenotype of fibroblasts. **(A–C)** Phenotyping of *ex vivo* and *in vitro* fibroblast populations. NFs **(A)**, CT26.WT- **(B)**, and 4T1-CAFs **(C)** were analyzed for their expression of CD39, CD87, Sca-1, CD95, Ly-6C, and CD44 before and after *in vitro* culture. Isolated fibroblasts were seeded at a density of 10^5^ cells/cm^2^. Analysis was performed when the cells reached a confluency of 80–90%, in these experiments at day 5 of cultivation. The percentage of cells expressing the respective marker is depicted. Data are reported as median and SEMs for three replicates from at least two independent experiments and statistical analysis was performed using the two-way ANOVA (^**^P < 0.005; ^****^P < 0.0001).

Comparison of CAFs and NFs revealed a differential expression of CD39 and CD87 *ex vivo*, next to additional CAF-related cell surface molecules. As many cell-based assays require a period of *in vitro* propagation, we next asked whether the CAF- and NF-related expression pattern of the most important candidate markers was preserved upon *in vitro* propagation. Normal fibroblasts were isolated from BALB/c mouse skin and fat pad, while 4T1-, and CT26.WT-derived CAFs were isolated from the respective tumor tissue. Cells were analyzed by flow cytometry *ex vivo* after immunomagnetic cell sorting. Isolated fibroblasts were plated on Matrigel-coated dishes at a density of 1 × 10^5^ cell/cm^2^. Cells were grown to a confluency of ~80–90%, usually reached at day 5–6, detached and analyzed again using flow cytometry. Comparison of the acutely isolated *ex vivo* and *in vitro* expression profiles of NFs showed that neither the characteristic expression of CD39 nor of Sca-1 was altered *in vitro*. Notably, a significant increase of NFs expressing CD87 (yet to a comparably low overall level of 20%), CD95, Ly-6C, and CD44 was observed *in vitro* (Figure 3A). These data suggested a change of marker expression based on the switch of the functional state upon cultivation toward a more cancer-associated phenotype. In contrast, expression profiles of CAFs were widely preserved *in vitro* (Figures 3B,C). Only CD44 was significantly reduced in 4T1-derived CAFs after *in vitro* propagation (Figure 3C), and also decreased in CT26.WT-derived CAFs (Figure 3B), indicating a trend for CD44 to decrease *in vitro*. For the other cell surface markers analyzed no significant induction or loss of surface marker expression was observed.

### Increased Expression of CD87, Sca-1, and Ly-6C in NFs Correlates With Their Proliferative Activity

**Figure 4:**
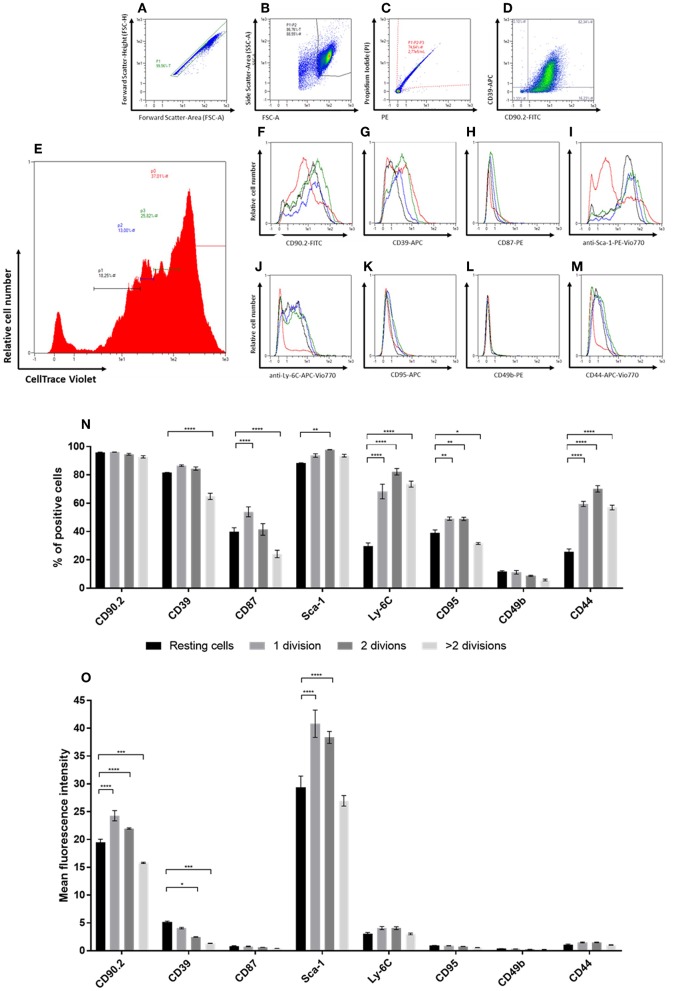
*In vitro* generational tracing assay. **(A–M)** Representative gating strategy and read-out for proliferation assay. First cells are gated in a FSC-A vs. FSC-H plot to exclude doublets **(A)**, followed by exclusion of debris on a FSC-A vs. SSC-A plot **(B)** and dead cell exclusion on a PE-A vs. PI-A plot **(C)**. The percentage of fibroblasts and the “NF phenotype” is confirmed in a FITC-A (CD90.2) vs. APC-A (CD39; **D**) plot. For proliferation analysis, populations with different numbers of cell divisions, represented by different peaks, are gated in a histogram indicating the CellTrace Violet fluorescence intensities **(E)**. The peak of cells with the highest CellTrace Violet fluorescence represented cells that did not divide, accordingly referred to as “resting” cells (red). The peaks with gradually lower CellTrace Violet fluorescence represented cell populations with increasing numbers of cell divisions, referred to as “1 division (green),” “2 divisions (blue),” and “>2 divisions (black),” respectively. Histograms of the populations with different numbers of cell divisions *in vitro* indicate the levels of expression for the different markers (CD90.2, CD39, CD87, Sca-1, Ly-6C, CD95, CD49b, and CD44; F-M). **(N,O)** The expression levels of the different markers within populations after distinct numbers of cell divisions are compared based on the percentage of cells expressing the respective marker **(N)** and expression level depicted as mean fluorescence intensities (MFIs) of the respective markers **(O)**. Proliferation analysis has been performed in triplicates in two independent experiments. Data are reported as mean and SEMs for three replicates and statistical analysis was performed using the two-way ANOVA (^*^P < 0.05; ^**^P < 0.005; ^***^P < 0.0005; ^****^P < 0.0001).

Upon analysis of fibroblasts *in vitro*, we found that cultured CAFs resembled the expression of candidates identified in the screening approach, while NFs showed a significantly increased expression of the CAF-associated markers CD44, CD87, CD95, and Ly-6C. As CAFs are described as a distinct type of activated fibroblasts and activated fibroblasts proliferate (e.g., during wound healing or inflammation processes), we hypothesized that those markers might be functionally linked to proliferation. To test this, we performed *in vitro* generational tracing assays. Normal fibroblasts were isolated from healthy BALB/c mice by immunomagnetic sorting, stained with a cell trace dye and plated at a density of 1 × 10^5^ cells/cm^2^. The cells were grown to a confluency of ~80–90% (at day 5 after seeding), before they were harvested and analyzed for the expression of CD39, CD44, CD49b, CD87, CD90.2, CD95, Ly-6C, and Sca-1, as well as for their amount of cell divisions by assessing the fluorescence intensity of the cell trace dye. Figures 4A–E shows the gating for proliferation analysis. The read out is shown in Figures 4E–M: populations of fibroblasts that had undergone different numbers of cell divisions were gated based on the fluorescence intensity of the cell trace dye (Figure 4E) and the marker expression was assessed for those populations (Figures 4F–M). Based on the amount of cells expressing the respective marker (Figure 4N) as well as the MFI among the respective subpopulations, a conclusion could be drawn whether the expression pattern correlated with proliferation.

The marker CD49b showed only low expression at comparable levels throughout the different populations and independent of the number of cell divisions. For the markers CD87, CD95, and CD44 an overall weak expression was detected as well (Figure 4O). However, the percentage of cells expressing those markers was markedly higher as compared to CD49b. Expression of CD90.2 was found among a comparable fraction of cells in all populations, independent of the number of cell divisions. Yet, the level of CD90.2 expression was significantly weaker in the fibroblast population of resting cells and cells that had undergone most cell doublings. The MFI and percentage of Sca-1-expressing cells detected within the population of resting fibroblasts was significantly lower than in cells that had undergone up to two cell divisions. However, cells that had undergone more than two cell divisions showed the same expression levels as resting fibroblasts. In comparison to resting cells, the number of cells expressing CD39 was found slightly increased in the proliferating populations, but was significantly lower in cells that had undergone most cell divisions. Additionally, the MFI of CD39 was significantly lower in cells with increasing numbers of cell divisions. The CAF-associated marker CD87 was found expressed on a subpopulation of NFs propagated *in vitro*. In contrast to the expression levels detected in CAFs *ex vivo* and *in vitro*, the MFI was found to be markedly lower in the NF subpopulations (MFI 0.4–0.9). The frequency of CD87-positive cells was significantly lower in resting fibroblasts compared to the cells that had undergone the first cell division. Interestingly, the population that had undergone most cell divisions showed even significantly lower fractions of CD87-expressing cells. *In vitro* propagated normal fibroblasts additionally showed differential expression of Ly-6C among the populations with different proliferation behavior. Upon proliferation, the fraction of Ly-6C-positive cells was found significantly increased, while the level of expression was not altered. The cell surface markers CD95 and CD44 showed similar expression profiles: resting cells showed significantly lower frequencies of cells expressing the respective marker, while the MFI showed no significant changes. Notably, cells that had undergone most cell divisions showed a significantly lower frequency of CD95-positive cells compared to resting cells. However, the overall expression level of CD95 and CD44 was very low (MFI 0.5–1 for CD95; MFI 1.0–1.6 for CD44).

Taken together, the expression levels of CD44, CD87, CD95, Sca-1, and Ly-6C positively correlated with activation, indicated by (initial) cell proliferation. However, the levels of CD44, CD87, and CD95 detected in NFs was still very low as compared to CAFs. Moreover, CD87 expression decreased after the second division, indicating that its expression is rather associated with activation than with proliferation *per se*. The expression level of CD90.2 was also found to be positively correlated with increased cell division, while CD39 showed a decrease in expression upon proliferation. The latter suggests an association of this marker with quiescence in NFs.

### “Healthy” NFs Are Marked by CD39 but Express CAF-Markers CD87 and CD49b in Tumor-Bearing Mice

**Figure 5:**
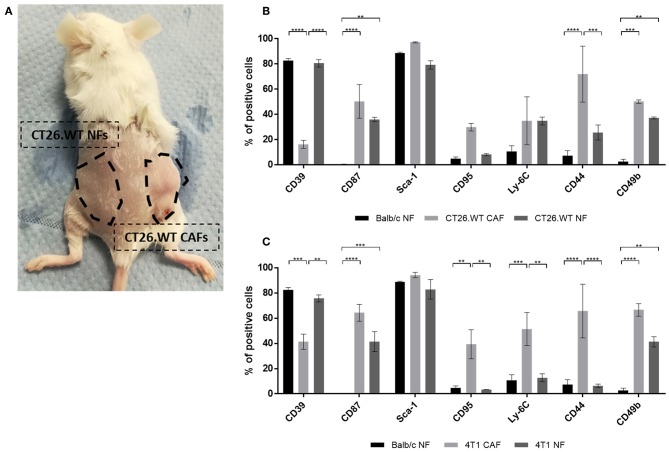
*Ex vivo* analysis of normal fibroblasts from tumor-bearing mice. **(A–C)** Comparison of NFs derived from tumor-bearing mice with “healthy” NFs and CAFs. Fibroblasts were isolated and analyzed *ex vivo* for their expression of CD39, CD87, Sca-1, CD95, Ly-6C, CD44, and CD49b. NFs from tumor-bearing mice were isolated from tumor-free skin and mammary fat pad of the flank where no tumor cells had been injected **(A)**, thus they have not been in direct contact with tumor cells. As healthy NF control population, fibroblasts were isolated from healthy BALB/c mice. The phenotype of NFs from tumor bearing mice was analyzed in CT26.WT **(B)** and 4T1 **(C)** mouse tumor model. The percentage of cells expressing the respective marker is depicted. Data are reported as median and SEMs for three replicates from at least two independent experiments and statistical analysis was performed using the two-way ANOVA (^**^P < 0.005; ^***^P < 0.0005; ^****^P < 0.0001).

After the analysis of NFs *in vitro* suggested a correlation of the CAF-associated markers with proliferation behavior, we sought to analyze NFs from tumor-bearing mice for their specific expression profiles. To the best of our knowledge, such data were completely missing to date. As tumors are thought to shape pre-metastatic niches in preparation of metastasizing (24, 25), we hypothesized that the expression of CAF-associated markers might be induced by such a mechanism leading to increased expression on NFs isolated from tumor-bearing mice. This would demonstrate an additional cancer-specific regulation mechanism of those markers. To test this hypotheses, skin and mammary fat pad of the flank without injected tumor cells was dissected and dissociated into a single cell suspension (Figure 5A). Fibroblasts were isolated from this “healthy tissue” and compared to CAFs isolated from the corresponding tumor as well as to normal fibroblasts isolated from healthy mice. Interestingly, the comparison revealed a mixed phenotype for NFs isolated from tumor-bearing mice (Figures 5B,C). In CT26.WT-derived tumor bearing mice, CD87 and CD49b were significantly overexpressed on fibroblasts (CT26.WT-NFs and CT26.WT-CAFs) in comparison to NFs from healthy mice (Figure 5B). In contrast, CD39 was significantly overexpressed only by CT26.WT-NFs and at the same level as found in NFs from healthy mice, while CD44 was significantly underrepresented in NFs from tumor mice and healthy mice in comparison to CAFs. Notably, CD95 expression in CT26.WT-NFs showed a trend toward the levels of “healthy” NFs and being underrepresented in comparison to CT26.WT-CAFs. Normal fibroblasts from 4T1 tumor-bearing mice showed the same expression patterns as CT26.WT-NFs for the markers CD39, CD87, CD44, and CD49b. In addition, CD95 and Ly-6C were significantly underrepresented in 4T1-NFs in comparison to 4T1-CAFs (Figure 5C).

Taken together, we identified CD39 as novel cell surface marker for normal fibroblasts in healthy as well as tumor-bearing mice. The expression of CD39 in NFs seemed to be negatively correlated with proliferation. In addition, our data suggested a cancer-associated expression of CD44, CD49b, CD87, CD95, and Ly-6C. Yet, they seemed to correlate with proliferation and accordingly were also found upregulated in proliferating NFs. Of note, CD49b and CD87 seemed to be upregulated in tumor-bearing mice in response to a tumor-associated systemic effect.

## Discussion

Many studies on CAFs rely on widely accepted markers for their identification but lack NFs as highly important control when describing and characterizing cancer-associated alterations (5, 15–20). Here, we demonstrated the identification of CAF- as well as NF-associated cell surface markers by systematically comparing these two subtypes of fibroblasts.

So far, mainly intracellular markers, such as αSMA (2, 26, 27), have been identified as suitable markers for distinguishing NFs and CAFs. Moreover, cell surface markers such as the PDGF receptors PDGFRα and PDGFRβ (27–29) are widely used to identify or isolate CAFs. Using a screening panel of 233 antibodies, the surface marker expression profiles of fibroblasts from healthy skin, healthy mammary fat pad, as well as subcutaneously induced 4T1- and CT26.WT-tumors were analyzed. Based on this screening, a panel of 62 markers was prepared and used for validation on isolated fibroblasts. Due to immunomagnetic sorting of the target cells, the data quality could be enhanced by removing non-fibroblasts, background noise, and unspecific signals. The markers EphA2, GARP, MHC class II, Rae-1a/b/g, CD80, CD87, CD106, CD123, and CD150 were detected to be expressed on 4T1- and CT26.WT-derived fibroblasts, whereas CD39 was detected specifically in normal fibroblasts. In addition, the expression patterns of CD95 and Ly-6C could be validated.

To the best of our knowledge, NF-associated markers that could facilitate identification of CAFs were lacking to date. We identified CD39 as the only cell surface marker specifically overexpressed in NFs based on these 233 candidates. CD39 is also known as ecto-nucleoside triphosphate diphosphohydrolase 1 (E-NTPDase1), and converts ATP into AMP when ATP has been released to the extracellular space (30, 31). It has been related to cancer mainly as tumor promoting molecule (32, 33) and is described to be expressed on immunomodulatory cells such as Tregs, functioning as immunosuppressive marker (34, 35). Moreover, its function is closely associated with the function of CD73 (30, 34). Yet, CD73 expression was not found to be correlated with CD39 in fibroblasts. Our findings suggested that CD39 is expressed at significantly lower levels in cancer-associated fibroblasts of BALB/c mice as compared to normal “healthy” fibroblasts of BALB/c dermal and mammary fat pad tissues. This was surprising with respect to the described role of CD39 in cancer (32–34).

Next to the NF-marker CD39, our screenings revealed several markers found significantly overexpressed among CAFs or subpopulations thereof. CD87, showing the highest specificity for CAFs, is the receptor for urokinase-type plasminogen activator (uPA), and is part of the plasminogen activation system (36). The serine protease uPA is capable of degrading extracellular matrix proteins and is involved in tissue remodeling processes that occur e.g., in wound healing (37–40). Furthermore, the uPA/uPAR interaction has been described to be involved in cancer, where matrix remodeling and MMPs have been known as key players in cancer progression for years (41–43). Therefore, it nicely matches with the finding that CD87 was overrepresented in populations of CAFs in BALB/c mice but hardly expressed in normal fibroblasts. Interactions of uPA and uPAR have been described to be involved in the process of metastasizing (37, 43, 44). It is possible that CD87 expression detected in NFs from tumor-bearing mice is associated to these observations and represents a premature sign of metastases.

Among the candidate markers correlating with the CAF phenotype, CD95 was identified in addition. CD95 is also known as death receptor Fas/APO-1 and has been associated with cancers in humans and mice for over a decade (45–50). Despite its contribution to the development of the immune system and regulation of immune responses, CD95 is reported to be involved in immune evasion of cancer cells and resistance to therapy (51–55). Interestingly, cancer cells apply different evasion mechanisms of CD95-induced apoptosis: reports showed that CD95 can be downregulated, but CD95 sensitivity can also be reduced by e.g., blocking of CD95 receptor and ligand binding (through soluble CD95L or soluble decoy receptor 3) or alteration of CD95 signaling pathway (51, 52, 54, 56–58). In this study, CD95 was found overrepresented in 4T1- and CT26.WT-CAFs *ex vivo*. Upon *in vitro* propagation, CD95 expression was unaffected in CAFs while NFs showed a significant upregulation of CD95 expression. Proliferation analysis of NFs showed a significant increase of CD95 expressing cells upon proliferation. It remains unclear whether CD95 signaling is altered in CAFs, comparable to the immune evasion mechanisms described in tumor cells. This could explain an inducibility in CD95 sensitive NFs but not in CD95-induced apoptosis-resistant CAFs.

Ly-6C is a characteristic molecule for myeloid cells (59, 60) and expressed on T cell subsets (61, 62), but was also found to be significantly overrepresented on cancer-associated fibroblasts *ex vivo*. The function of Ly-6C remains widely unknown, yet, it is mainly associated with the immune system, and inflammatory events (63–65). We identified Ly-6C as characteristic marker for CAFs, which is in line with current literature (66–68). Ly-6C has been introduced as cell surface marker characteristic for a subpopulation of activated (cancer-associated) fibroblasts, termed inflammatory CAFs [iCAFs; (66–68)]. However, our data suggested Ly-6C to be a general fibroblast marker upregulated upon activation or proliferation and generally overexpressed in CAFs. This observation was only possible because in our study healthy NFs were always used as matched controls, rather than using putative progenitor cells (67, 68) or basing the observations on the expression of CAF-associated markers alone (5, 15–20).

Much like CD95 and Ly-6C, the glycoprotein CD44 was significantly overrepresented in CAFs isolated from 4T1, and CT26.WT tumors. This marker has been related to different processes in cancer for decades (66, 69–71). Interestingly, this dramatically changed upon *in vitro* propagation and significant overrepresentation was found in NFs subsequently, yet, at markedly lower overall levels than observed for CAFs. Like Ly-6C, CD44 expression was found upregulated in proliferating NFs while quiescent NFs did not show a CD44 expression *in vitro*. These data suggested a correlation of CD44 expression to proliferative activity, matching reports of CD44 expression in activated fibroblasts involved in inflammation and wound healing processes (72, 73). In contrast, CD44 levels in CAFs propagated *in vitro* were decreased, pointing at other functions of CD44 in CAFs. In a study of the association of CD44-expressing CAFs and cancer cell stemness (5), it was shown that stromal CD44 expression was predominantly found in hypoxic areas of tumor tissue. Thus, if CD44 in CAFs is associated with or even induced by hypoxic conditions, it would explain that CD44 expression in CAFs propagated under normoxic *in vitro* conditions will downregulate CD44, despite actively proliferating. The authors of this study also tested this hypothesis and cultured CAFs under hypoxic conditions. Surprisingly, they saw even a decrease of CD44 expression of CAFs cultured under hypoxic conditions as compared to CAFs under normoxia. However, in their setting, CD44 expression was induced under hypoxic and hyponutritional conditions.

Interestingly, the widely used CAF-associated cell surface molecules PDGFRα and PDGFRβ (27–29) were not identified as CAF-specific candidates. We found PDGFR expression among fractions of NFs, yet, to a lower extent compared to CAFs (data not shown). This suggested that PDGFRs might not represent the best markers for identification and isolation of CAFs from solid tumors.

Most reports on CAF function for example use *in vitro* assays to study CAFs and their interaction with other cell types (5, 16, 68, 74, 75). However, cultivation could potentially affect the behavior of cells as reflected by altered expression of characteristic markers among others. Notably, regulation of expression patterns could also have functional implications. According to our results depicted in Figure 3, upon cultivation CAFs widely preserved their specific expression patterns of markers identified before. On the contrary, NFs preserved expression of their characteristic marker CD39, but upregulated CAF-associated markers upon *in vitro* propagation. In light of the proliferation assay results, the CAF-associated markers are suggested to be surrogate markers for activation and proliferation among fibroblasts. Thus, those markers could be constantly expressed in CAFs which are thought to be highly activated fibroblasts and the expression is not affected *in vitro*, while expression of activation-associated markers is upregulated in NFs upon propagation and culture conditions that induce proliferation.

We found CD39 expression as being a characteristic feature of NFs, while expression of CAF-associated markers seemed to be also induced upon proliferation. Accordingly, we expected low expression of CAF-associated markers in NFs isolated from healthy tissues of tumor-bearing mice. Indeed, the markers CD44, CD95, and Ly-6C were expressed at low levels in NFs from tumor-bearing mice, suggesting a mainly activation- and proliferation-associated role and regulation. However, the markers CD49b, and CD87 were significantly upregulated in NFs from tumor-bearing mice. This demonstrated an independent, disease-associated role of those markers. Moreover, this showed that tumors can have a systemic effect on fibroblasts, even before they have metastasized. These findings could pave the way for identification of fibroblasts and markers in pre-metastatic lesions. For our study, we employed widely used cell line-induced tumor models. In future studies, these findings will have to be evaluated for general applicability using genetically or chemically induced tumor models.

Taken together, by employing a workflow optimized to immunomagnetically isolate fibroblasts from solid tissues and using appropriate healthy control cell populations, we have identified CD39 as novel cell surface marker for NFs as well as CD44, CD49b, CD87, CD95, and Ly-6C as CAF-associated markers. Our data suggested an association of the CAF markers, most strikingly CD87 and Ly-6C, with fibroblast activation and proliferation. Furthermore, the widely known stem cell-associated marker Sca-1 and the expression level of CD90.2 correlated with fibroblast proliferation in our assays. We demonstrated that induction of CD49b and CD87 expression is triggered in NFs from healthy tissues by systemic cancer induced effects in tumor-bearing mice.

## Data Availability

All datasets generated for this study are included in the manuscript and/or the **Supplementary Files**.

## Ethics Statement

This study was carried out in accordance with the recommendations of European and German guidelines for the care of laboratory animals. The protocol was approved by the ethical committee on animal care and use in North Rhine-Westphalia (approval number: 84_02.04.2014.A325).

## Author Contributions

DA, AB, SW, and OH conceived and designed the experiments. DA, AL, and UH performed the experiments. DA, AL, and OH analyzed the data. DA, AB, and OH wrote the manuscript.

### Conflict of Interest Statement

DA, AL, UH, SW, AB, and OH are or were full time employees of Miltenyi Biotec GmbH.
