# Supplementary material for: CD49b, CD87, and CD95 Are Markers for Activated Cancer-Associated Fibroblasts Whereas CD39 Marks Quiescent Normal Fibroblasts in Murine Tumor Models
Source: Front Oncol. 2019 Aug 5;9:716. doi: 10.3389/fonc.2019.00716 (PMC6690267; doi:10.3389/fonc.2019.00716)
Supplement: Supplementary file 1 [file Data_Sheet_1.docx]

Supplementary Material

## Supplementary Figures


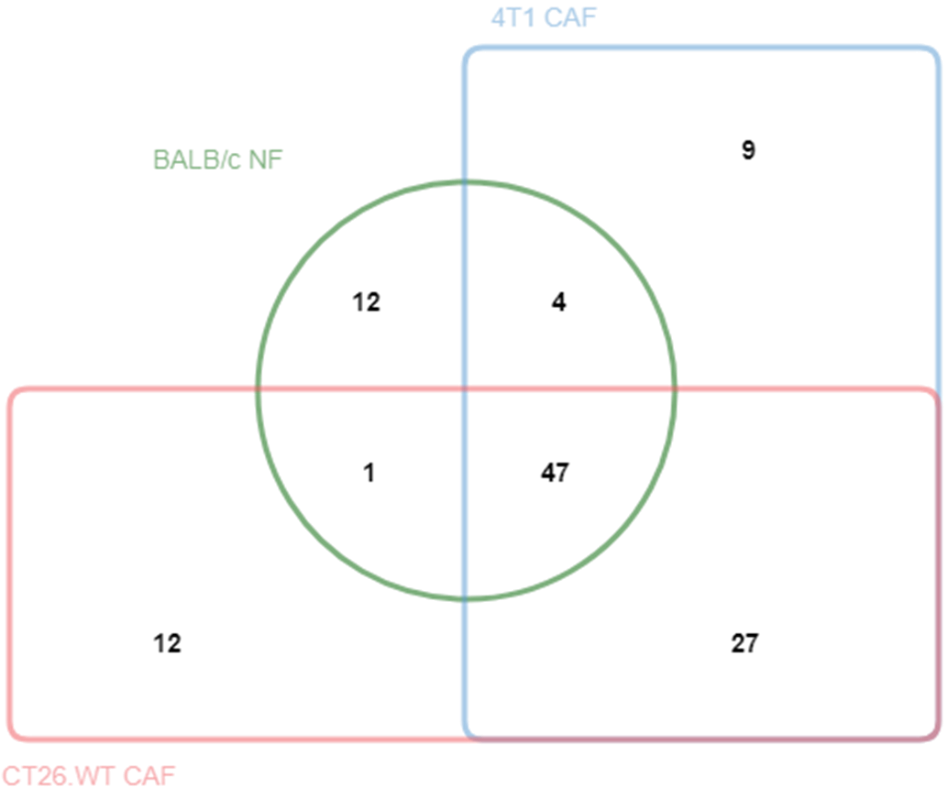


**Supplementary figure 1: Identification of cell surface markers differentially expressed among normal and cancer-associated fibroblasts**

Identification of differentially expressed cell surface markers in normal fibroblasts (BALB/c NF), CT26.WT-CAFs and 4T1-CAFs. All markers found to be expressed on the fibroblasts populations (as defined by the expression pattern CD90.2^pos^ CD45^neg^ Ter119^neg^ respectively) are depicted as Edwards-Venn diagram (Bardou, Mariette, Escudie, Djemiel, & Klopp, 2014). Of all the markers found to be expressed in fibroblast populations, 47 markers were not specific for either fibroblast population. In contrast, 12 surface markers were exclusively expressed on NFs (green), 9 markers were exclusively expressed on 4T1-CAFs (blue) and 12 markers were exclusively expressed on CT26.WT-CAFs (red). Additionally, 27 markers were expressed in both CAF population but not in NFs, thus representing candidate markers for cancer-associated fibroblasts in general.


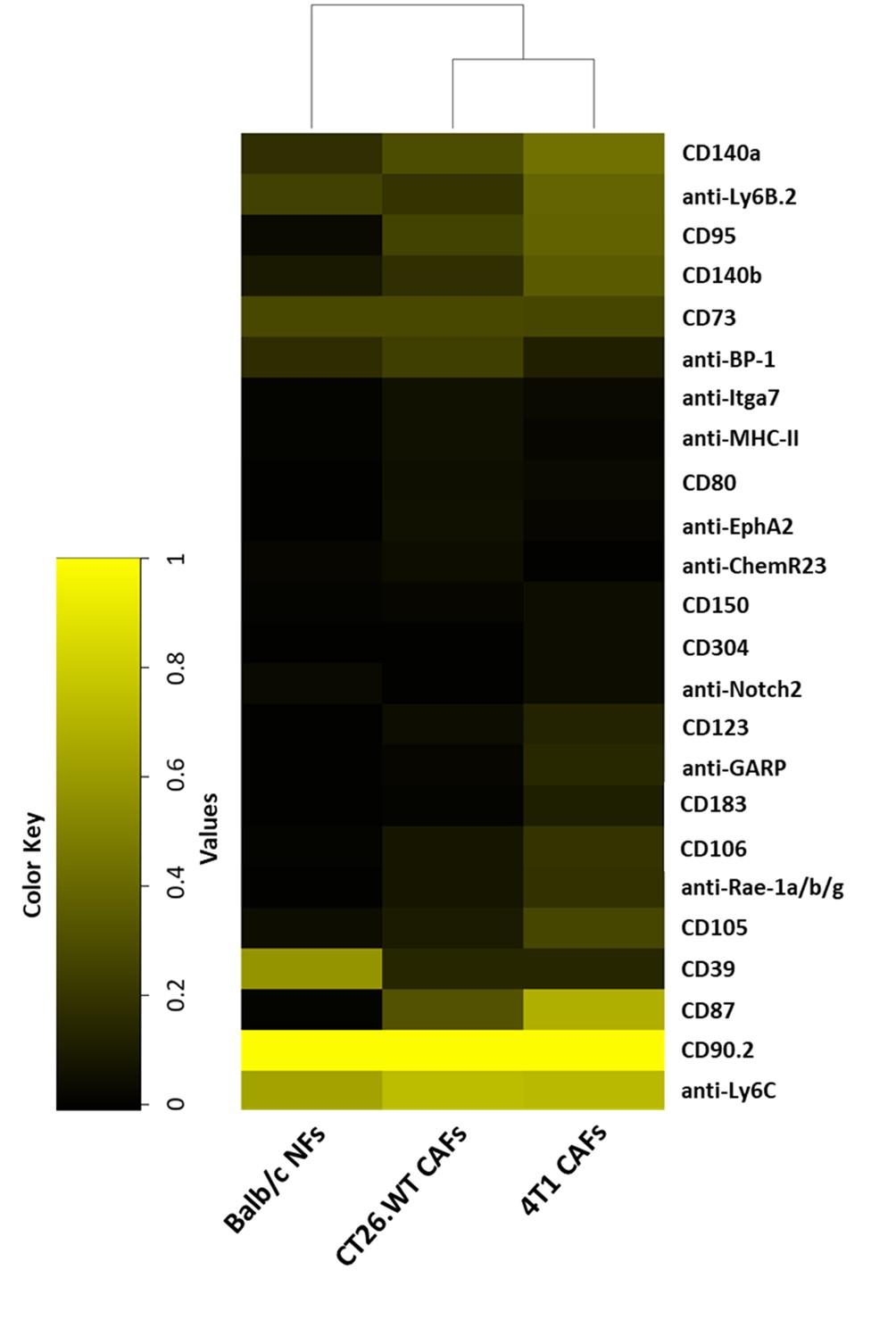


**Supplementary figure 2: Hierarchical clustering of validation data**

Validation of the 62 marker panel by flow cytometry on isolated normal fibroblasts (BALB/c NF), CT26.WT-CAFs and 4T1-CAFs *ex vivo*. In the heatmap, all markers expressed by at ≥ 3 % of fibroblasts are depicted. The expression of 24 markers was validated. Complete Euclidean clustering of the three fibroblast populations reveals a closer relation of 4T1- and CT26.WT-CAFs, NFs cluster more distinctly.

## Supplementary Tables

**Table S1:** List of antibodies used for the screening experiments. All antibodies used were PE-conjugated.

| **Specificity** | **Miltenyi Biotec order number** | **Clone** |
| --- | --- | --- |
| anti-A2B5 | 130-093-581 | 105HB29 |
| anti-ACSA-2 | 130-109-864 | IH3-18A3 |
| anti-B7-H4 | 130–106–412 | REA392 |
| anti-BP-1 | 130–102–190 | 6C3 |
| anti-ChemR23 | 130–106–902 | REA461 |
| anti-Clec9a | 130–102–362 | 7H11 |
| anti-DCAR-1 | 130–102–396 | SKa5–3D5 |
| anti-DCIR-2 | 130–102–188 | 33D1 |
| anti-Dectin-1 | 130–102–284 | REA154 |
| anti-DLL-4 | 130–102–449 | HMD4–1 |
| anti-DO11.10 TCR | 130–102–198 | KJ1–26 |
| anti-DR-3 | 130–107–126 | REA475 |
| anti-Embigin | 130–108–010 | REA501 |
| anti-EOMES | 130–102–916 | REA116 |
| anti-EphA2 | 130–109–139 | REA579 |
| anti-F4/80 | 130–102–943 | REA126 |
| anti-FcR Ia | 130–102–351 | MAR-1 |
| anti-FR-4 | 130–102–455 | TH6 |
| anti-Galectin-3 | 130–101–315 | M3/38 |
| anti-Galectin-9 | 130–102–236 | RG9–35.7 |
| anti-GARP | 130–102–182 | REA139 |
| anti-GFAP | 130–105–326 | REA335 |
| anti-GITR (CD357) | 130–102–428 | DTA–1 |
| anti-GLAST | 130–095–821 | ACSA-1 |
| anti-GR-1 | 130–102–426 | RB6–8C5 |
| anti-H2Kb/SIINFEKL | 130–102–180 | 25–D1.16 |
| anti-H2Kd/H2Dd | 130–107–894 | REA527 |
| anti-H2Kk | 130–102–394 | H100–27.R55 |
| anti-H60a | 130–108–820 | REA556 |
| anti-IgD | 130–102–459 | 11–26c.2a |
| anti-IgDa | 130–107–133 | REA484 |
| anti-IgG2ab | 130-095-877 | X-57 |
| anti-IgM | 130-095-908 | X-54 |
| anti-Integrin a7 | 130–102–716 | 3C12 |
| anti-Integrin b7 | 130–106–440 | REA441 |
| anti-Interferon-gR b chain | 130–105–671 | REA381 |
| anti-Jagged 2 | 130–102–316 | HMJ2-1 |
| anti-KLRG-1 | 130–102–408 | 2F1 |
| anti-LPAM-1 | 130–106–950 | REA457 |
| anti-LT-bR | 130–106–593 | REA416 |
| anti-Ly49A | 130–102–418 | A1 |
| anti-Ly49C/F/I/H | 130–106–112 | 14B11 |
| anti-Ly49C/I | 130–102–754 | REA253 |
| anti-Ly49D | 130–102–416 | 4E5 |
| anti-Ly49E/F | 130-102-697 | REA218 |
| anti-Ly49F | 130–104–292 | HBF-719 |
| anti-Ly49G | 130–102–417 | AT-8 |
| anti-Ly49G2 | 130–102–332 | 4D11 |
| anti-Ly49H | 130–102–704 | REA241 |
| anti-Ly49I | 130–106–108 | YLI-90 |
| anti-Ly6B.2 | 130–102–370 | REA115 |
| anti-Ly6C | 130–102–391 | 1G7.G10 |
| anti-Ly6G | 130–102–392 | 1A8 |
| anti-MEK1 | 130–101–064 | REA155 |
| anti-Mer | 130–107–478 | REA477 |
| anti-MHC-II | 130–102–186 | M5/114.15.2 |
| anti-MHC-II I-Ab | 130–107–941 | REA528 |
| anti-MHC-II I-Ak | 130–109–272 | REA610 |
| anti-MHC-II I-Ek | 130–107–820 | REA510 |
| anti-Microfold (M) Cell | 130–102–150 | NKM 16–2–4 |
| anti-Nk1.1 | 130–102–400 | PK136 |
| anti-NKG2 A/C/E | 130–105–568 | 20d5 |
| anti-Notch 1 | 130–102–304 | 22E5 |
| anti-Notch 2 | 130–102–369 | HMN2–35 |
| anti-Notch 4 | 130–102–360 | HMN4–14 |
| anti-O4 | 130–095–887 | O4 |
| anti-O4 REA | 130–109–152 | REA576 |
| anti-PIR-A/B | 130–106–961 | REA472 |
| anti-Plexin B2 | 130–106–335 | REA445 |
| anti-Prominin-1 | 130–102–210 | MB9–3G8 |
| anti-PSA-NCAM | 130–093–274 | 2-2B |
| anti-QA-1B | 130–104–219 | 6A8.6F10.1A6 |
| anti-QA-2 | 130–107–907 | REA523 |
| anti-Rae-1a/b/g | 130–109–011 | REA578 |
| anti-Sca-1 | 130–106–424 | REA422 |
| anti-Siglec F | 130–102–274 | ES22–10D8 |
| anti-Siglec H | 130–102–261 | 551.3D3 |
| anti-SSEA-1 | 130–104–936 | REA321 |
| anti-Syndecan 4 | 130–109–830 | REA640 |
| anti-TCR b | 130–104–813 | REA318 |
| anti-TCR g/d | 130–104–012 | GL3 |
| anti-TCR Va 11.1,11.2 | 130–106–940 | REA464 |
| anti-TCR Va 3.2 (b,c) | 130–106–004 | REA395 |
| anti-TCR Vb10 | 130–109–936 | REA651 |
| anti-TCR Vb11 | 130–109–928 | REA657 |
| anti-TCR Vb14 | 130–110–051 | REA645 |
| anti-TCR Vb17a | 130–109–934 | REA647 |
| anti-TCR Vb3 | 130–109–850 | REA646 |
| anti-TCR Vb8.1/8.2 | 130–109–025 | REA585 |
| anti-TCR Vd4 | 130–105–675 | REA372 |
| anti-Ter119 | 130–102–336 | Ter-119 |
| anti-TIGIT | 130–108–233 | REA536 |
| anti-Tim-3 | 130–109–447 | RMT3–23 |
| anti-Tom22 | 130–107–697 | 1C9–2 |
| CD100 | 130–104–716 | REA322 |
| CD101 | 130–104–303 | REA301 |
| CD103 | 130–102–567 | 2E7 |
| CD104 | 130–106–923 | REA456 |
| CD105 | 130–102–548 | MJ7/18 |
| CD106 | 130–104–712 | 429 (MVCAM.A) |
| CD107a | 130–102–219 | 1D4B |
| CD107b | 130–106–290 | M3/84 |
| CD115 | 130–102–554 | AFS98 |
| CD117 | 130–102–542 | 3C11 |
| CD119 | 130–104–934 | REA189 |
| CD11b | 130–091–240 | M1/70.15.11.5 |
| CD11c | 130–102–545 | N418 |
| CD120b | 130–104–697 | REA228 |
| CD122 | 130–102–569 | TM–β1 |
| CD123 | 130–102–583 | REA114 |
| CD124 | 130–102–710 | REA235 |
| CD125 | 130–105–982 | REA343 |
| CD126 | 130–105–848 | D7715A7 |
| CD126 | 130–109–566 | REA620 |
| CD127 | 130–102–551 | A7R 34 |
| CD131 | 130–102–588 | REA193 |
| CD132 | 130–106–130 | REA403 |
| CD134 REA | 130–109–741 | REA625 |
| CD135 | 130–102–564 | A2F10 |
| CD137 | 130–102–568 | 17B5–1H1 |
| CD137L | 130–105–853 | TKS–1 |
| CD138 | 130–102–580 | REA104 |
| CD140a REA | 130–109–736 | REA637 |
| CD140b REA | 130–109–867 | REA634 |
| CD144 | 130–102–737 | REA225 |
| CD146 | 130–102–319 | ME–9F1 |
| CD15 | 130-104-936 | REA321 |
| CD150 | 130–104–639 | REA299 |
| CD151 | 130–109–005 | REA561 |
| CD152 | 130–102–570 | UC10–4B9 |
| CD153 | 130–102–575 | RM153 |
| CD154 | 130–102–467 | MR1 |
| CD155 | 130–107–807 | REA519 |
| CD16/CD32 | 130–107–039 | REA377 |
| CD166 | 130–105–446 | REA370 |
| CD169 | 130–104–953 | REA197 |
| CD171 | 130–102–243 | 555 |
| CD178 | 130–102–590 | MFL3 |
| CD18 | 130–104–018 | M18/2 |
| CD180 | 130–102–589 | RP/14 |
| CD183 | 130–102–275 | CXCR3–173 |
| CD184 | 130–102–282 | REA107 |
| CD185 | 130–102–288 | REA215 |
| CD19 | 130–102–598 | 6D5 |
| CD192 | 130–108–722 | REA538 |
| CD193 | 130–102–325 | REA122 |
| CD195 | 130–105–142 | REA354 |
| CD196 | 130–103–816 | REA277 |
| CD197 | 130–102–756 | 4B12 |
| CD199 | 130–102–317 | CW–1.2 |
| CD1d | 130–102–603 | 1B1 |
| CD2 | 130–102–622 | RM2-5 |
| CD201 | 130–106–585 | 1560 |
| CD204 | 130–102–328 | REA148 |
| CD205 | 130–102–231 | NLDC–145 |
| CD207 | 130–102–202 | caa8–28H10 |
| CD209b | 130–106–330 | REA125 |
| CD21/CD35 | 130–102–440 | 7E9 |
| CD22 | 130–102–610 | Cy34.1 |
| CD223 | 130–102–578 | C9B7W |
| CD229 | 130–102–767 | REA273 |
| CD23 | 130–102–611 | B3B4 |
| CD24 | 130–102–732 | M1/69 |
| CD244.1 | 130–107–936 | REA524 |
| CD244.2 | 130–105–988 | REA388 |
| CD25 | 130–102–593 | 7D4 |
| CD252 | 130–102–582 | RM134L |
| CD253 | 130–102–562 | N2B2 |
| CD255 | 130–102–273 | MTW–1 |
| CD256 | 130–105–346 | REA347 |
| CD262 | 130–102–565 | MD5–1–3C2 |
| CD266 | 130–104–280 | ITEM–4 |
| CD267 | 130–102–721 | 8F10 |
| CD268 | 130–102–553 | 7H22–E16 |
| CD269 | 130–108–300 | REA550 |
| CD269 | 130–108–300 | REA550 |
| CD270 | 130–102–771 | REA275 |
| CD272 | 130–102–689 | REA224 |
| CD273 | 130–102–280 | MIH37 |
| CD275 | 130–102–283 | HK5.3 |
| CD278 | 130–100–795 | REA192 |
| CD278 | 130–100–795 | REA192 |
| CD279 | 130–102–299 | HA2–7B1 |
| CD282 | 130–099–016 | REA109 |
| CD283 | 130–102–759 | 11F8 |
| CD284 | 130–102–561 | MTS510 |
| CD30 | 130–103–942 | mCD30.1 |
| CD300a | 130–109–036 | REA573 |
| CD301a | 130–109–216 | REA581 |
| CD304 | 130–105–658 | REA380 |
| CD307a | 130–108–683 | REA566 |
| CD309 | 130–102–559 | Avas12 |
| CD31 | 130–102–608 | 309 |
| CD310 | 130–102–216 | AFL4 |
| CD314 | 130–102–728 | CX5 |
| CD317 | 130-102-293 | JF05-1C2.4.1 |
| CD326 | 130–102–265 | caa7–9G8 |
| CD335 (anti-NKp46) | 130-102-395 | 29A1.4.9 |
| CD352 | 130–109–860 | 13G3 |
| CD354 | 130–102–252 | REA191 |
| CD371 | 130–109–263 | REA594 |
| CD38 | 130–102–607 | 90.4 |
| CD4 | 130-102-619 | GK1.5 |
| CD40 | 130–102–599 | FGK45.5 |
| CD41 | 130–105–872 | MWReg30 |
| CD43 | 130–102–594 | L11 |
| CD45 | 130–102–596 | 30F11 |
| CD45.1 | 130–102–499 | A20 |
| CD45.2 | 130–102–498 | 104-2 |
| CD45R | 130–102–292 | RA3–6B2 |
| CD45RA | 130–102–526 | 14.8 |
| CD45RB | 130–102–525 | C363–16A |
| CD47 | 130–102–617 | REA170 |
| CD48 | 130–102–609 | HM48-1 |
| CD49b | 130–108–174 | REA541 |
| CD49d | 130–102–555 | R1-2 |
| CD5 | 130–102–620 | 53-7.3 |
| CD6 | 130–104–594 | REA311 |
| CD61 | 130–102–628 | 2C9.G2 (HMβ3–1) |
| CD62E | 130–105–467 | REA369 |
| CD62L | 130–102–543 | MEL14–H2.100 |
| CD62P | 130–105–536 | REA344 |
| CD64 | 130–103–808 | REA286 |
| CD68 | 130–102–614 | FA-11 |
| CD69 | 130–103–946 | H1.2F3 |
| CD73 | 130–102–616 | TY/11.8 |
| CD79b | 130–105–833 | HM79–12 |
| CD83 | 130–104–474 | REA304 |
| CD84 | 130–102–618 | REA212 |
| CD85k | 130-099-808 | REA141 |
| CD86 | 130–102–604 | PO3.3 |
| CD8a | 130–102–595 | 53-6.7 |
| CD90.2 | 130–102–489 | 30–H12 |

**Table S2:** Overview of the validation panel. All antibodies used were PE-conjugated.

| **Specificity** | **Miltenyi Biotec order number** | **Clone** |
| --- | --- | --- |
| CD90.2 | 130-102-489 | 30-H12 |
| anti-B7-H2 (CD275) | 130-116-446 | REA990 |
| anti-B7-H4 | 130-106-412 | REA392 |
| anti-BP-1 | 130-116-579 | REA988 |
| anti-ChemR23 | 130-106-902 | REA461 |
| anti-EphA2 | 130-109-139 | REA579 |
| anti-Galectin-9 | 130-118-774 | REA1069 |
| *anti-GARP* | 130-102-921 | REA139 |
| anti-GLAST | 130-118-344 | ACSA-1 |
| anti-H2Kb/SIINFEKL | 130-116-794 | REA1002 |
| anti-IFN gamma Rb | 130-105-671 | REA381 |
| anti-Integrin a7 | 130-103-355 | 3C12 |
| anti-Jagged 2 | 130-102-316 | HMJ2-1 |
| anti-LPAM-1 | 130-106-950 | REA457 |
| *anti-Ly49 e/f* | 130-103-336 | REA218 |
| anti-Ly49G | 130-117-275 | REA1022 |
| anti-Ly6B.2 | 130-102-857 | REA115 |
| anti-Ly-6C | 130-111-916 | REA796 |
| anti-MHC-II | 130-102-896 | M5/114.15.2 |
| *anti-Notch2* | 130-102-854 | HMN2-35 |
| anti-Notch 4 | 130-102-360 | HMN4-14 |
| anti-Rae-1a/b/g | 130-109-011 | REA578 |
| anti-SSEA-1 | 130-117-801 | REA321 |
| anti-Tim-3 (CD366) | 130-102-415 | RMT3-23 |
| CD100 | 130-104-716 | REA322 |
| CD105 | 130-102-819 | MJ7/18 |
| CD106 | 130-104-712 | 429 (MVCAM.A) |
| *CD119* | 130-104-934 | REA189 |
| CD123 | 130-102-583 | REA114 |
| *CD124* | 130-103-349 | REA235 |
| CD134 (OX40) | 130-102-572 | OX-86 |
| CD135 | 130-102-564 | A2F10 |
| CD137L | 130-105-853 | TKS-1 |
| CD140a REA | 130-109-736 | REA637 |
| CD140b REA | 130-109-867 | REA634 |
| *CD150 (SLAM)* | 130-104-680 | REA299 |
| CD152 | 130-116-390 | REA984 |
| CD153 | 130-102-575 | RM153 |
| CD16/CD32 | 130-102-429 | 93 |
| CD223 (LAG-3) | 130-102-578 | C9B7W |
| CD252 (OX40L) | 130-115-997 | REA960 |
| CD256 (APRIL) | 130-105-387 | REA347 |
| CD257 (BAFF) | 130-111-028 | REA767 |
| CD262 | 130-102-565 | MD5-1-3C2 |
| CD266 (FN-14) | 130-104-280 | ITEM-4 |
| CD267 (TACI) | 130-103-360 | 8F10 |
| CD270 (HVEM) | 130-103-410 | REA275 |
| CD272 (BTLA) | 130-103-328 | REA224 |
| CD279 | 130-111-800 | REA802 |
| CD304 (BDCA-4) | 130-105-705 | REA380 |
| CD309 (VEGFR-2) | 130-098-905 | ES8-20E6 |
| CD310 (VEGFR-3) | 130-112-583 | REA834 |
| CD354 (TREM-1) | 130-103-067 | REA191 |
| CD39 | 130-114-250 | REA870 |
| CD40 | 130-102-599 | FGK45.5 |
| *CD41* | 130-105-872 | MWReg30 |
| CD70 | 130-102-605 | FR 70 |
| CD73 | 130-111-331 | REA778 |
| CD80 | 130-102-613 | 16-10A1 |
| CD86 | 130-102-604 | PO3.3 |
| CD87 | 130-109-855 | REA630 |
| CD95 | 130-106-906 | REA453 |
